# The Effect of the Addition of Chokeberry Fiber on the Quality of Sheep's Milk Fermented by *Lactobacillus rhamnosus* and *Lactobacillus acidophilus*

**DOI:** 10.1155/2021/7928745

**Published:** 2021-07-11

**Authors:** K. Szajnar, M. Pawlos, A. Znamirowska

**Affiliations:** Department of Dairy Technology, University of Rzeszow, Cwiklinskiej St. 2D, Rzeszow 35-601, Poland

## Abstract

The global market for sheep's milk and its products is increasing due to higher demand for cheese and traditional dairy products, and as a novelty, sheep's milk is an ingredient in infant formulas and nutraceuticals. The aim of this study was to determine the properties of fermented sheep's milk, which combines probiotic benefits with increased dietary fiber content. The influence of the applied dose of chokeberry fiber on the growth of living cells of *Lactobacillus acidophilus* and *Lactobacillus rhamnosus* in fermented sheep's milk was also evaluated. Sheep milk with the addition of 0% (control sample), 1.5%, and 3.0% chokeberry fiber was fermented by two different probiotic monocultures *L. acidophilus* and *L. rhamnosus*. In the fermented milk, pH value, syneresis (%), color, and texture were determined. Furthermore, the microbiological analysis and an organoleptic evaluation were performed. With the increasing dose of chokeberry fiber, the pH value decreased already before the fermentation process. After fermentation, the milk's pH values with fiber were still lower than those in the control sample. Presumably, more acidic metabolites were produced by *L. acidophilus*, causing a more intense reduction of the pH value than *L. rhamnosus* both in control milk and in milk with the addition of fiber. The addition of chokeberry fiber affected the stimulation of the growth of both types of bacteria. In the milk sample without fiber addition, a more significant number of viable cells were counted for *L. acidophilus* by 0.5 log CFU g^−1^ more than the milk fermented with *L. rhamnosus*. Furthermore, in milk fermented by *L. acidophilus* with 1.5% chokeberry fiber (LA1), the number of viable bacterial cells was higher than that in milk fermented by *L. rhamnosus* with the same addition of fiber (LR1). However, in sample LA3, the number of viable bacterial cells was lower than that in sample LR3. Tested fermented sheep milk met the Recommendation of the International Dairy Federation's requirements regarding the number of live bacterial cells for dairy probiotics. The addition of chokeberry fiber increased syneresis in each fermented milk group, regardless of the bacteria used for fermentation. Moreover, the use of fiber caused a significant reduction in brightness *L*∗, an increase of red color, and a decrease of yellow color. Milk fermented with *L. acidophilus* was characterized by a harder gel, compared to their analogues fermented with *L. rhamnosus*. The addition of fiber intensified the sour taste and the taste of the additive in both types of fermented sheep milk.

## 1. Introduction

The demand for sheep's milk is growing on the global market due to its valuable properties, such as higher protein content, favourable fat content, and better source of functional bioactive peptides than cow's milk. Nowadays, China is the world's largest producer of sheep's milk. In the Claeys et al. [1] study, the horse, donkey, and sheep milk was compared with human milk, and it was indicated that sheep milk is a suitable alternative to breast milk and infant formula.

Sheep's milk production in the world is around 10.4 Mt [2]. The consumption of sheep milk has grown worldwide over the years and is expected to reach the level 2.7 Mt by 2030 [2]. The rapid expansion of sheep milk is caused by higher interest in cheese and traditional dairy products and its recent use as an ingredient in infant formula and nutraceutical products [3]. The global functional food market is developing rapidly, and probiotic products represent a potential area of growth worldwide. Moreover, probiotics, prebiotics, and synbiotics (a combination of probiotics and prebiotics) have become more and more popular [4].

The promising probiotic strains include bacteria of the genus *Lactobacillus*, *Bifidobacterium*, and *Enterococcus*. *Lactobacillus* GG and a variant of *L. casei* ssp. *rhamnosus* are the most studied probiotics and shown to be efficacious in reducing the severity and duration of diarrhoea [5].

The use of fruit fibers or their pulp may also improve probiotic bacteria's survival during refrigerated storage due to their protective effect and positively changing buffering capacity [6]. However, the addition of fruit pulp or fruit fiber during the production of probiotic yogurt can be a challenge, mainly because of the acidity of the fruit and the possible presence of antimicrobial constituents and changes in organoleptic properties and texture, and should be considered in the production process [7].

In turn, the use of chokeberry fiber as a prebiotic in the production of milk fermented by probiotic monocultures may contribute to the stimulation or limitation of their growth. What is more, chokeberry is a rich source of polyphenol compounds, carotenoids, vitamins, and bioelements. These compounds are responsible for the antioxidant, anticancer, improved circulation, radioprotective, and anti-inflammatory effects of chokeberry [8–11].

Previous studies indicate that 1-3% fiber addition can be applied to fermented milk, possibly more when using inulin. Nastaj and Gustaw [12] used 1%, 2%, and 3% prebiotic addition to yoghurt. Sah et al. [13] applied 1% fiber addition in the form of pineapple peel powder to probiotic yoghurt. Capela et al. [14] reported that the prebiotic “Raftilose®P95” added at 1.5% (*w*/*v*) to fermented milk improved the viability of combined selected organisms (*Lactobacillus acidophilus*, *Lactobacillus casei*, *Lactobacillus rhamnosus*, and *Bifidobacterium* ssp.) by 1.42 log during four weeks of storage at 4°C. Conversely, Guven et al. [15] found that the addition of 1% inulin improved the properties of low-fat yoghurt made from skimmed milk, which was comparable to yoghurt made from whole milk. A similar study showed that the firmness and brightness of low-fat yoghurt were improved when 2% inulin was added [16]. Oliveira et al. [17] found that yoghurt firmness increased when 4% inulin was added as a prebiotic in yoghurt. Furthermore, Helal et al. [18] also described that the apparent viscosity of yoghurt increased when inulin was added to yoghurt up to 2%.

Therefore, the aim of this study was to investigate the properties of fermented sheep's milk, which combines probiotic benefits with increased dietary fiber content. The effect of the applied dose of chokeberry fiber on the growth of living cells of *L. acidophilus* and *L. rhamnosus* in fermented sheep's milk was also evaluated.

## 2. Materials and Methods

### 2.1. Materials

Raw sheep's milk (Lacaune sheep breed, Seweryn's Farm, Podkarpackie, Poland) with a chemical composition of protein 5.20 ± 0.12%, fat 6.00 ± 0.57%, lactose 5.00 ± 0.10%, total solid 16.02 ± 0.42%, and pH 6.71 ± 0.02 was obtained from a local farm in Podkarpackie region, Poland. The chokeberry fiber was purchased from Natura Wita sp. z o.o. (Pińczów, Poland). The commercial probiotic starters *L. rhamnosus* Pen E/N Oxy® and *L. acidophilus* LA-5® were obtained from Chr. Hansen, Denmark.

Fermented milk manufacture was prepared according to the Kavaz and Bakirci [19] method with modifications.

Sheep's milk was divided into six batches, to which chokeberry fiber was added in varying amounts, and groups were coded:

LA: control milk fermented by *L. acidophilus*

LA1: milk with 1.5% chokeberry fiber fermented by *L. acidophilus*

LA3: milk with 3.0% chokeberry fiber fermented by *L. acidophilus*

LR: control milk fermented by *L. rhamnosus*

LR1: milk with 1.5% chokeberry fiber fermented by *L. rhamnosus*

LR3: milk with 3.0% chokeberry fiber fermented by *L. rhamnosus*

Milk with chokeberry fiber was homogenized using a homogenizer (Nuoni GJJ-0.06/40, Zhejiang, China), with a pressure of 20 MPa at 60°C. Then, the milk was pasteurized at 85°C for 30 minutes and cooled to 37°C.

After pasteurization and cooling, milk samples were divided into two batches. The first batch of milk with chokeberry fiber was inoculated with a single starter culture of *L. acidophilus* LA-5®. The control sample for this group was milk without the addition of fiber. The second batch of milk with chokeberry fiber was inoculated with *L. rhamnosus* Pen E/N Oxy®, and the control sample for this group was milk without the addition of fiber.

Each batch of milk was inoculated with a previously activated starter culture according to the Szajnar et al. [20] method (in the form of a bulk activated at 40°C for 5 h; after 5 h, inoculum consisted of log 9 CFU g −1 of bacteria, which was added to the milk in the amount of 5%). Inoculated milk was stirred and poured into 100 mL plastic cups. Packages were closed on a Desktop Semiautomatic Sealing Machine (China), sealed with heat-sealing foil, and fermented at 37°C for 10 h. Inoculated milk was stirred and poured into 100 mL plastic cups and fermented at 37°C for 10 h. The final products were cooled to 5°C ± 0.5°C (Cooled Incubator ILW 115, POL-EKO Aparatura, Poland). The experiment was repeated three times. Fermented milk was evaluated after the first day of cold storage (5°C ± 0.5°C).

### 2.2. Physicochemical Properties

#### 2.2.1. pH

The pH determination was performed with a pH meter (FiveEasy, Mettler Toledo, 105 Switzerland) using an electrode InLab®Solids Pro-ISM (Mettler Toledo, Switzerland). In milk, after the addition of fiber before fermentation, three measurements of pH values were taken for each group, whereas in fermented milk, pH was tested in a total of 90 samples (15 × 6 = 90).

#### 2.2.2. Syneresis (%)

Syneresis was determined using the centrifugal method as the released whey over the original weight according to the Santillán-Urquiza et al. [21] method with modifications using Laboratory Refrigerated Centrifuge LMC-4200R (Biosan SIA, Latvia) (10 g of fermented milk was transferred into 50 mL plastic tube and centrifuged for 10 min, 5°C). In fermented milk, syneresis was analyzed in a total of 90 samples (15 × 6 = 90).

#### 2.2.3. Color

The color was analyzed with a colorimeter (the Precision Colorimeter, Model NR 145, China) using the CIELAB system. The image brightness was determined with the parameter *L*∗ and chromaticity using *a*∗, *b*∗, *C*, *h*. Before testing, the device was calibrated on a white reference standard [22]. In fermented milk, color was measured in a total of 90 samples (15 × 6 = 90).

### 2.3. Texture Analyses

The TPA test determined the texturometric profile using a CT3 Texture Analyzer (Brookfield, USA) with TexturePro CT (Brookfield, USA) software. The sample dimensions were cylinder 66 mm × 33.86 mm, and the temperature of the sample was 8°C. The test was performed using the acrylic probe TA 3/100 and the following settings: distance 15 mm, contact load 0.1 N, and measurement speed 1 mm/s [23]. In fermented milk, texture was examined in a total of 90 samples (15 × 6 = 90).

### 2.4. Organoleptic Parameters

Organoleptic properties of potentially probiotic fermented milk were evaluated on a 9 cm linear scale, nonstructured. The following descriptors were studied: consistency, milky-creamy taste, sour taste, a taste of additives, sweet taste, off-taste, sour odor, odor of additives, and off-odor [14]. Organoleptic parameters were evaluated by the trained team: 10 women and 10 men (ages 20-40). The samples of fermented milk were assessed on a 9-point scale, with markings at both ends. The left end denoted the least intense and the least characteristic feature: sandy and grainy consistency, brightest color, and impalpable milky-creamy taste, sour taste, sweet taste, off-taste, and additive taste, and the right end denoted the most characteristic feature: smooth texture, the darkest color, and the most intense milky-creamy taste, sour taste, sweet taste, off-taste, and chokeberry fiber taste [24].

### 2.5. Microbiology Analysis

The number of microorganisms, *L. acidophilus* and *L. rhamnosus*, was estimated according to the method by Lima et al. [25], and the method description partly reproduces their wording. Viable counts of *L. acidophilus* and *L. rhamnosus* were evaluated using MRS agar (Biocorp, Poland) and incubated anaerobically at 37°C for 72 h. The results were expressed as log CFU g^−1^.

### 2.6. Statistical Analysis

The obtained results were given as the mean and standard deviation and were calculated statistically using the Statistica v. 13.1 software (StatSoft, USA). A two-way ANOVA was used to investigate the overall effect type of bacteria∗fiber on fermented milk properties. The significance of differences between the averages was estimated with Tukey's test (*P* ≤ 0.05).

## 3. Results and Discussion

### 3.1. Physicochemical Properties: pH

**Figure 1:**
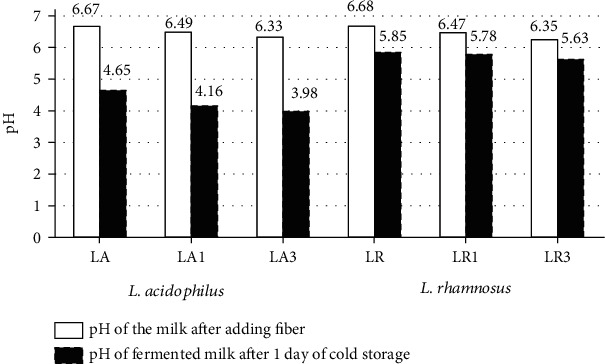
The pH values in milk after the addition of chokeberry fiber before fermentation and after the 1^st^ day of cold storage.

Figure 1 illustrates the pH value of the control milk sample after the addition of chokeberry fiber.

With an increasing dose of chokeberry fiber, the pH value decreased in milk before the fermentation process. The addition of 1.5% of fiber resulted in a reduction of pH by 0.18-0.21 and the addition of 3.0% of fiber up to 0.33-0.34 units compared to the control sample. According to Szopa et al. [11], in dried chokeberry, phenolic acids (hydroxycinnamic acid, caffeic acid), depsides (chlorogenic acid, neochlorogenic acid), organic acids (including citric acid and malic acid), and vitamin C may be found, which lowers the pH value before fermentation of milk.

The analysis of the pH of milk after fermentation showed that the pH value depends on the dose of chokeberry fiber and the type of bacterial strain used for milk fermentation. Presumably, more acidic metabolites were produced by *L. acidophilus*, causing a more intense reduction of the pH value than *L. rhamnosus* both in control milk and in milk with the addition of fiber. Moreover, it was indicated that after fermentation, the dependence of ever-lower pH values was maintained with increasing the dose of chokeberry fiber. The two-way ANOVA (Table 1) showed that the pH value after fermentation was significantly influenced by the type of bacteria and the addition of fiber, as well as the interactions between these factors.

### 3.2. Microbiology Analysis

**Figure 2:**
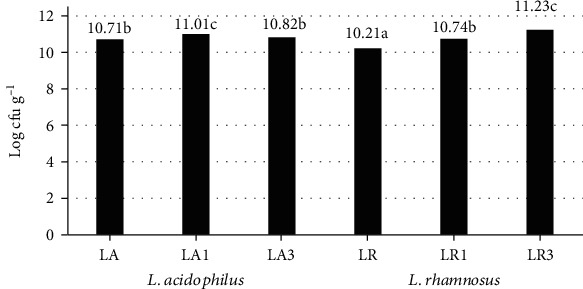
Viable counts in fermented milk with chokeberry fiber (log CFU g^−1^) *L. acidophilus*, *L. rhamnosus*. Values are means ± S.D. for *n* = 15. a–c: mean values between fermented milk with chokeberry fiber denoted by different letters differ statistically significantly (*P* ≤ 0.05).

Figure 2 shows the number of cells of two types of bacteria in fermented sheep's milk depending on the amount of addition of chokeberry fiber. In milk, without fiber addition, the increased number of viable cells was counted for *L. acidophilus* by 0.5 log CFU g^−1^ more compared to the milk fermented by *L. rhamnosus*. According to the International Dairy Federation's Recommendation, the probiotic products should contain at least 7 log CFU g^−1^ of lactic acid bacteria [26]. Tested fermented sheep's milk met these requirements.

The addition of chokeberry fiber contributed to the stimulation of the growth of both types of bacteria. The addition of 1.5% chokeberry fiber to sheep's milk had the most beneficial effect on the number of *L. acidophilus* cells. In the present case, enhancing the fiber dose to 3% increased the number of *L. acidophilus* cells only by 0.1 log CFU g^−1^ compared to the LA control sample. Therefore, there was not shown a significant difference. The addition of chokeberry fiber to sheep's milk significantly stimulated the growth of *L. rhamnosus*. It was observed that with the increase in the dose of chokeberry fiber, the number of *L. rhamnosus* cells gradually increased. In milk with 1.5% chokeberry fiber addition, the number of *L. rhamnosus* cells increased by 0.53 log CFU g^−1^. In a sample with 3% of fiber, the number of bacterial cells increased by 1.02 log CFU g^−1^ compared to the control sample LR.

The optimal pH value for *L. rhamnosus* growth is in the range of 6.4 to 6.9 [27], and the minimum pH may range from 4.4 to 3.4, depending on the buffer capacity of the medium [28]. The sheep's milk indicated pH values before fermentation and after the fermentation process oscillated in that range. Furthermore, the growth requirements for *L. rhamnosus* include folic acid, riboflavin, niacin, pantothenic acid, and calcium [29], which are present in sheep's milk. *L. rhamnosus* is facultative heterofermentative bacteria (lactobacilli group 2), which converts hexoses to L (+) lactic acid, following the Embden-Meyerhof route. Due to aldolase and phosphoketolase, pentoses are also fermented.

A possible explanation for the stimulatory effect of fiber, mainly phenolic compounds, on bacteria's growth would be that some microorganisms can use these compounds as substrates. Certain species of bacteria, such as lactobacilli, can metabolize phenolic compounds during growth, and therefore, the polyphenols provide energy to the cell. Also, phenolic compounds, except for having a positive effect on bacterial metabolism, can increase the consumption of nutrients such as sugars [30–32]. According to Hervert-Hernández et al. [33], polyphenols extracted from grape marc may act as a *L. acidophilus* growth-stimulating factor. Moreover, Zhu et al. [34] reported that anthocyanins might promote the growth of Lactobacillus due to the prebiotic activity of anthocyanins and anthocyanin monomers.

### 3.3. Syneresis (%)

The lowest syneresis of acid gel was demonstrated in the control milk sample fermented with *L. rhamnosus* (Table 2), and the rate of whey leakage was 3.0% higher in the control group fermented with *L. acidophilus.* The addition of chokeberry fiber increased syneresis in each group, regardless of the bacteria used for fermentation. The addition of 1.50% chokeberry fiber resulted in an increase in syneresis by 7.40% in the LA1 group and by 14.85% in the LR1 group compared to their control analogues. However, the 3.0% addition of fiber increased syneresis, respectively, 5.41% and 22.14%.

The ability to expand and thus absorb water depends on the type of fiber, its granulation, and heat treatment. Chokeberry fiber consists of cellulose (33.14 g/100 g d.m.), hemicellulose (32.08 g/100 g d.m.), lignins (23.03 g/100 g d.m.), and pectins (7.52 g/100 g d.m.) [35]. These proportions of the constituent fractions of chokeberry fiber indicate its poor solubility and ability to absorb water. Furthermore, it contains procyanidins (tannins), which are well known for their ability to react with proteins as they precipitate albumin and other proteins from solutions, which may also enhance syneresis [36].

### 3.4. Color

Table 2 shows the color parameters showing the range of color changes depending on the chokeberry fiber amount introduced. The control sheep's milk LA and LR were characterized by a high brightness value *L*∗, indicating white color. The values of the parameters *a*∗and *b*∗ indicated the contribution of green and yellow hues to the color development of the control samples. The introduction of fiber caused a significant decrease in the brightness of *L*∗ and an increase in the proportion of red color and decreased the proportion of yellow color. The color changes were attributed to the presence of polyphenols in chokeberry fiber.

According to Wolski et al. [37], in chokeberry fruit, 24.3% of all polyphenolic compounds are anthocyanins, which cause color changes depending on the pH value. In the acidic environment, they are redder, while in the neutral environment more violet [38, 39]. According to Oszmiański and Lachowicz [40], 24.7 g/100 dry weight of polyphenolic compounds was determined in dry chokeberry fruit powder. The main polyphenolic compounds in chokeberry products were anthocyanins > procyanidin polymers > phenolic acids > flavonols > flavan‐3‐ols > flavanones. According to Oszmiański and Lachowicz [40], the main PC compounds identified in chokeberry powders were anthocyanins, which constituted ~50% of total polyphenols.

The lower pH values reported in milk fermented by *L. acidophilus* LA1 and LA3 also resulted in a higher proportion of red color than in their analogues fermented by *L. rhamnosus* LR1 and LR3, which showed higher pH values. The addition of chokeberry fiber changed the hue (*h*) of fermented sheep's milk.

The structure of anthocyanin molecules has a significant effect on the hue, intensity, and stability of the color. Increasing the number of hydroxyl groups in the ring shifts the absorption maximum to the long-wavelength direction and causes a color change from orange through red to violet, whereas replacing hydroxyl groups with methoxyl groups reverses this trend.

Two-way ANOVA only showed no effect of type of bacteria conducting fermentation on color brightness. The other parameters characterizing color were significantly affected by the studied factors (type of bacteria and fiber) and their interactions.

Table 3 shows the texture parameters of milk fermented by *L. acidophilus* and *L. rhamnosus* with different addition of chokeberry fiber. Hardness is the essential component of texture and is defined as the force that must be applied to ensure a specific deformation of the fermented milk gel [41].

Harder gels characterized milk fermented by *L. acidophilus* compared to their analogues fermented by *L. rhamnosus*. The reason for the lower hardness of milk fermented by *L. rhamnosus* might be due to its lower acidity (pH on the first day of storage).

The addition of chokeberry fiber and the quantity of its dose influenced the hardness of milk significantly. The addition of 1.5% chokeberry fiber increased hardness by 0.09 N in the LA1 sample and by 0.15 N in the LR1 sample compared to controls. Increasing the fiber dose to 3.0% resulted in an increase in the fermented milk's hardness and by 0.31 N in samples LA3 and LR3 compared to the control.

Adhesiveness indicates strong binding and affects fermented milk's structural integrity [42, 43]. Cohesiveness is associated with stronger gel composition [44]. The adhesiveness of the LA and LR control samples differed significantly, up to 1.39 mJ. The lower adhesiveness was observed in milk fermented by *L. rhamnosus*, and the addition of fiber increased the adhesiveness. However, the addition of 3.0% fiber did not increase adhesiveness to a level comparable to the LA sample. The addition of 1.5% fiber in LA1 milk also increased adhesiveness compared to control LA, and an increase in the fiber dose to 3.0% in LA3 milk resulted in an adhesiveness increased by up to 1.15 mJ.

The analysis of the influence of both factors (type of bacteria and fiber) showed no significant influence of their interactions on the fermented milk's cohesiveness and springiness. A two-way ANOVA also indicated that interactions between types of bacteria∗fiber did not significantly affect cohesiveness and springiness.

### 3.5. Organoleptic Parameters

**Figure 3:**
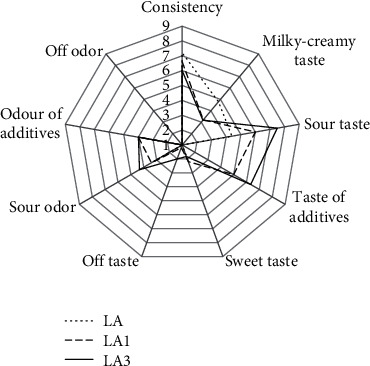
Effect of added chokeberry fiber on organoleptic parameters of fermented milk by *L. acidophilus.*

**Figure 4:**
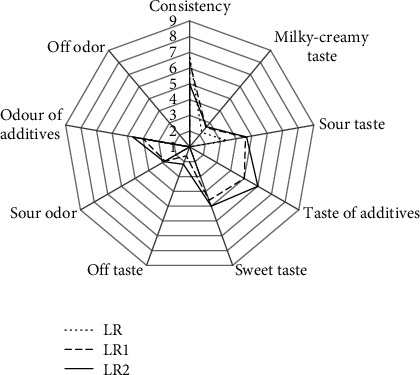
Effect of added chokeberry fiber on organoleptic parameters of fermented milk by *L. rhamnosus.*

Figures 3 and 4 show the organoleptic parameters of fermented milk. Sheep's milk fermented by *L. rhamnosus* had a looser consistency than milk with *L. acidophilus*, which was also indicated by the results that determined the samples' hardness. The milky-creamy taste was less intense in the samples with fiber than in the controls. The sweet and sour taste of fermented milk was significantly correlated with the type of bacteria performing the fermentation. Milk fermented by *L. acidophilus* had a more intense sour taste, whereas milk fermented by *L. rhamnosus* had a sweeter taste. The addition of chokeberry fiber and increasing its dose to 3.0% intensified the perception of sour taste and additives' taste and smell. According to Wolski et al. [37], chokeberry products contain organic acids and vitamin C, which also intensified the sour taste. Furthermore, acidity could be suppressed by sweetness [45].

Assessors noted a mild off-taste in the samples with fiber, with perception increased at the 3.0% chokeberry fiber dose. The presence of off-taste in fermented milk with chokeberry fiber is a consequence of the fiber's procyanidin content. According to Szajdek and Borowska [46] and Wawer [47], procyanidins, otherwise known as tannins, derived from chokeberry, affect products' sensory properties by providing a bitter and astringent taste.

The dried chokeberry contains approximately 6.30 mg of tannin/100 g. According to Oszmiański and Lachowicz [40], procyanidin polymers are the second group of polyphenolic compounds in chokeberry products and constitute 40% of the total polyphenol content. They constitute an essential group of health-promoting compounds and are responsible for the strong taste of chokeberry products. Procyanidin oligomers exhibit a high affinity to proteins, thereby causing their denaturation. Such an effect is perceptible during chokeberry products' consumption [48].

An important compound identified in chokeberry products was eriodictyol-glucuronide, belonging to the group of flavanones, representing ~0.01% of the total polyphenol contents. It is mainly responsible for chokeberry fruits' bitter taste [9].

## 4. Conclusions

Sheep's milk provides an attractive food matrix for the growth of lactobacilli. In this study, probiotic sheep's fermented milk was successfully obtained with the addition of a prebiotic in the form of chokeberry fiber. The milk with the fiber addition was characterized by a more intense additive taste and acidic flavour.

The addition of fiber to sheep's milk before fermentation stimulated the growth of *L. rhamnosus* and *L. acidophilus*. Moreover, it was shown that the intensity of sweet taste and sour taste was correlated with the type of bacteria that carried out the fermentation. Sheep's milk fermented by *L. rhamnosus* and *L. acidophilus* monocultures with the addition of polyphenol-rich fiber is desirable in the diet for protection against cardiovascular disease. Supplementation of chokeberry fiber to fermented sheep's milk may be interesting for the functional food market due to the beneficial effects of this prebiotic fiber rich in polyphenols.

## Figures and Tables

**Table 1:** Analysis of variance (ANOVA) *P* values on the effects of type of bacteria and fiber addition on pH, syneresis, color parameters: *L*∗, *a*∗, *b*∗, *C*, *h*, hardness, adhesiveness, cohesiveness, springiness, consistency, milky-creamy taste, sour taste, taste of additives, sweet taste, off-taste, sour odor, odor of additives, and off-odor of fermented milk.

Properties	Type of bacteria *P* values	Fiber *P* values	Type of bacteria∗fiber *P* values
pH	0.0000↑	0.0000↑	0.0000↑
Syneresis	0.0000↑	0.0000↑	0.0000↑
*L*∗	0.3074 n.s.	0.0000↑	0.0000↑
*a*∗	0.0000↑	0.0000↑	0.0000↑
*b*∗	0.0000↑	0.0000↑	0.0183↑
*C*	0.0000↑	0.0000↑	0.0000↑
*h*	0.0000↑	0.0000↑	0.0000↑
Hardness	0.0000↑	0.0000↑	0.1521 n.s.
Adhesiveness	0.0000↑	0.0002↑	0.9208 n.s.
Cohesiveness	0.2404 n.s.	0.5519 n.s.	0.5781 n.s.
Springiness	0.1964 n.s.	0.5074 n.s.	0.4489 n.s.
Consistency	0.7862	0.0946 n.s.	0.6655 n.s.
Milky-creamy taste	0.2513 n.s.	0.8528 n.s.	0.1027 n.s.
Sour taste	0.0000↑	0.0156↑	0.0861 n.s.
Taste of additives	0.7312 n.s.	0.0000↑	0.9641 n.s.
Sweet taste	0.0000↑	0.1590 n.s.	0.0479↑
Off-taste	0.5972 n.s.	0.0098↑	0.6339 n.s.
Sour odor	0.2125 n.s.	0.8095 n.s.	0.6378 n.s.
Odor of additives	0.6960 n.s.	0.0000↑	0.8480 n.s.
Off-odor	0.9547 n.s.	0.5116 n.s.	0.5116 n.s.

Type of bacteria∗fiber = interaction; ↑ indicates significant effect *P* ≤ 0.05; n.s.: no significant effect.

**Table 2:** Color and syneresis of milk fermented by *L. acidophilus*/*L. rhamnosus* with chokeberry fiber.

Properties	LA	LA1	LA3	LR	LR1	LR3
Syneresis (%)	13.77 ± 1.56^a^	21.17 ± 1.30^b^	19.18 ± 0.54^b^	10.52 ± 1.28^a^	25.37 ± 1.88^c^	32.66 ± 0.10^d^
*L*∗	95.42 ± 0.79^c^	63.95 ± 0.89^b^	54.02 ± 1.76^a^	93.33 ± 0.47^c^	65.32 ± 1.16^b^	56.34 ± 2.39^a^
*a*∗	−1.01 ± 0.12^a^	13.83 ± 0.34^d^	17.63 ± 0.34^e^	−1.13 ± 0.04^a^	8.73 ± 0.45^b^	11.17 ± 0.72^c^
*b*∗	9.83 ± 0.36^d^	1.72 ± 0.14^b^	0.62 ± 0.45^a^	10.93 ± 0.56^e^	4.02 ± 0.52^c^	2.53 ± 0.52^b^
*C*	9.88 ± 0.37^a^	13.93 ± 0.32^c^	17.64 ± 0.35^d^	10.16 ± 0.39^b^	10.62 ± 0.52^b^	11.47 ± 0.60^b^
*h*	95.89 ± 0.60^e^	7.10 ± 0.72^b^	2.03 ± 1.43^a^	95.82 ± 0.21^e^	24.68 ± 2.62^d^	11.69 ± 0.94^c^

Values are the mean ± S.D. for *n* = 15 × 6 = 90. a–e: mean values in lines denoted by different letters differ statistically significantly (*P* ≤ 0.05).

**Table 3:** Parameters of texture of milk fermented by *L. acidophilus*/*L. rhamnosus* with chokeberry fiber.

Properties	LA	LA1	LA3	LR	LR1	LR3
Hardness (N)	1.06 ± 0.03^d^	1.15 ± 0.04^e^	1.37 ± 0.01^f^	0.59 ± 0.01^a^	0.74 ± 0.01^b^	0.90 ± 0.05^c^
Adhesiveness (mJ)	1.70 ± 0.35^bc^	2.13 ± 0.21^cd^	2.85 ± 0.21^d^	0.31 ± 0.17^a^	0.63 ± 0.48^ab^	1.33 ± 0.06^b^
Cohesiveness (mJ)	0.49 ± 0.13^a^	0.45 ± 0.11^a^	0.43 ± 0.11^a^	0.70 ± 0.12^a^	0.67 ± 0.11^a^	0.49 ± 0.11^a^
Springiness (mm)	13.12 ± 0.12^a^	13.16 ± 0.14^a^	13.17 ± 0.09^a^	13.08 ± 0.12^a^	13.09 ± 0.14^a^	13.09 ± 0.03^a^

Values are the mean ± S.D. for *n* = 15 × 6 = 90. a–f: mean values in lines denoted by different letters differ statistically significantly (*P* ≤ 0.05).

## Data Availability

All the numerical data used to support the findings of this study are available from the corresponding author upon request.
